# Supplementary material for: Bidirectional ATP-driven transport of cobalamin by the mycobacterial ABC transporter BacA
Source: Nat Commun. 2024 Mar 23;15:2626. doi: 10.1038/s41467-024-46917-1 (PMC10960864; doi:10.1038/s41467-024-46917-1)
Supplement: Supplementary file 1 — Supplementary Information [file 41467_2024_46917_MOESM1_ESM.pdf]

Supplementary Information

Bidirectional ATP-driven transport of cobalamin by the mycobacterial ABC transporter BacA

Mark Nijland[1], Solène N. Lefebvre[1], Chancievan Thangaratnarajah[1,2], Dirk J. Slotboom[1,*]

Affiliation

[1]Faculty of Science and Engineering, Groningen, Biomolecular Sciences and Biotechnology, Membrane Enzymology Group, University of Groningen, 9747 AG, Groningen, The Netherlands. [2]Sosei Heptares, Steinmetz Building, Granta Park, Great Abington, Cambridge, CB21 6DG, United Kingdom.

*email: d.j.slotboom@rug.nl

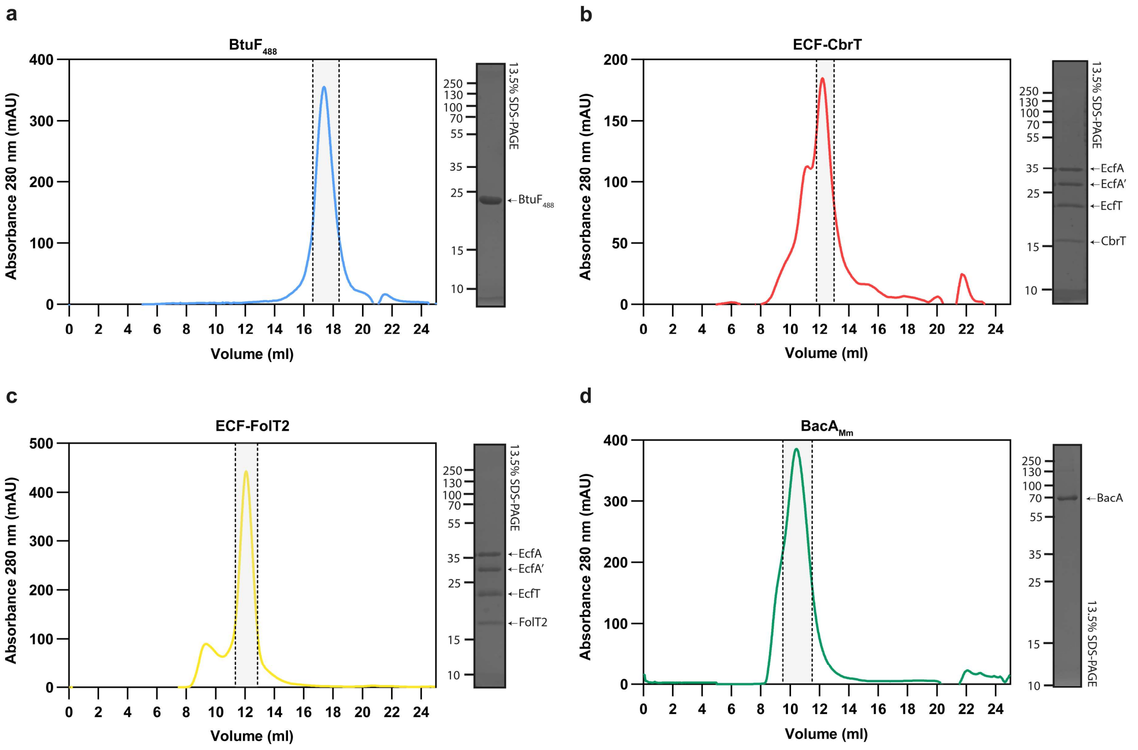

Supplementary Figure 1. Protein purifications. a-d Size exclusion profiles of proteins separated on a Superdex 200 increase 10/300 GL size exclusion column. The peak fractions that were used for biochemical assays are indicated by black dotted lines. The protein standards and samples that were analyzed on Coomassie stained 13.5% SDS-PAGE gels are indicated. **a** $BtuF_{488}$, **b** ECF-CbrT, **c** ECF-FolT2, **d** $BacA_{Mm}$. The uncropped SDS-PAGE gel images are shown in Supplementary Fig. 6.

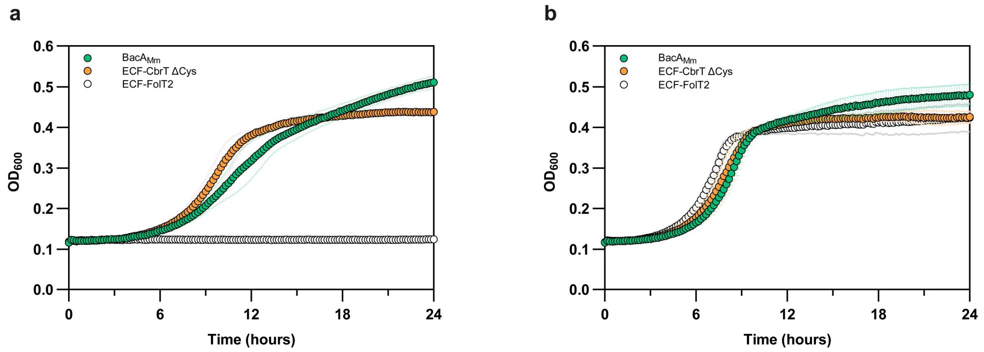

Supplementary Figure 2: Cobalamin-dependent growth assay. a Growth assay in M9 minimal medium supplemented with 1 nM cobalamin using the cobalamin-deficient *E. coli* ΔFEC strain expressing either ECF-FolT2 (white), ECF-CbrT (orange) or BacA$_{Mm}$ (green). A positive control for growth in presence of methionine is shown in panel **b**. **b** Similar growth assay in M9 minimal medium supplemented with 50 µg ml^{-1} methionine using the cobalamin-deficient *E. coli* ΔFEC strain expressing either ECF-FolT2 (white), ECF-CbrT (orange) or BacA$_{Mm}$ (green). Data was obtained from three biological repeats each containing three technical repeats. The data is presented as the mean with error bars indicating the standard deviation calculated from all individual data points (n=9).

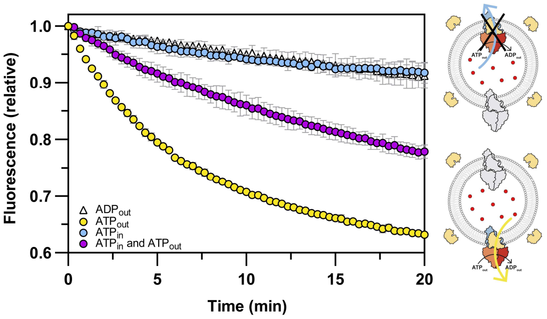

Supplementary Figure 3: Fluorescence based transport assay for cobalamin. Release of cobalamin from proteoliposomes reconstituted with the cobalamin transporter ECF-CbrT. Proteoliposomes were loaded with 65 µM cobalamin and the sensor BtuF$_{488}$ was added to the external buffer with a concentration of 25 nM. Transport activity was monitored in the presence of 10 mM external Mg-ATP (yellow circles), 10 mM internal Mg-ATP (blue circles), 10 mM Mg-ATP on both sides (purple circles) and 10 mM external Mg-ADP (white triangles). Cartoons on the right side of the graph explain the experimental setup. The liposome membrane is shown as grey circle, the sensor in the external buffer in yellow, cobalamin as red dots, the active ECF-CbrT population in colors, and the inactive population in grey. Export and import directions are indicated by blue and yellow arrows respectively. The black cross indicates that export is not catalysed by ECF-CbrT. The data were obtained from biological duplicates containing each technical triplicates. The data is presented as the mean with error bars indicating the standard deviation calculated from all individual data points (n=6).

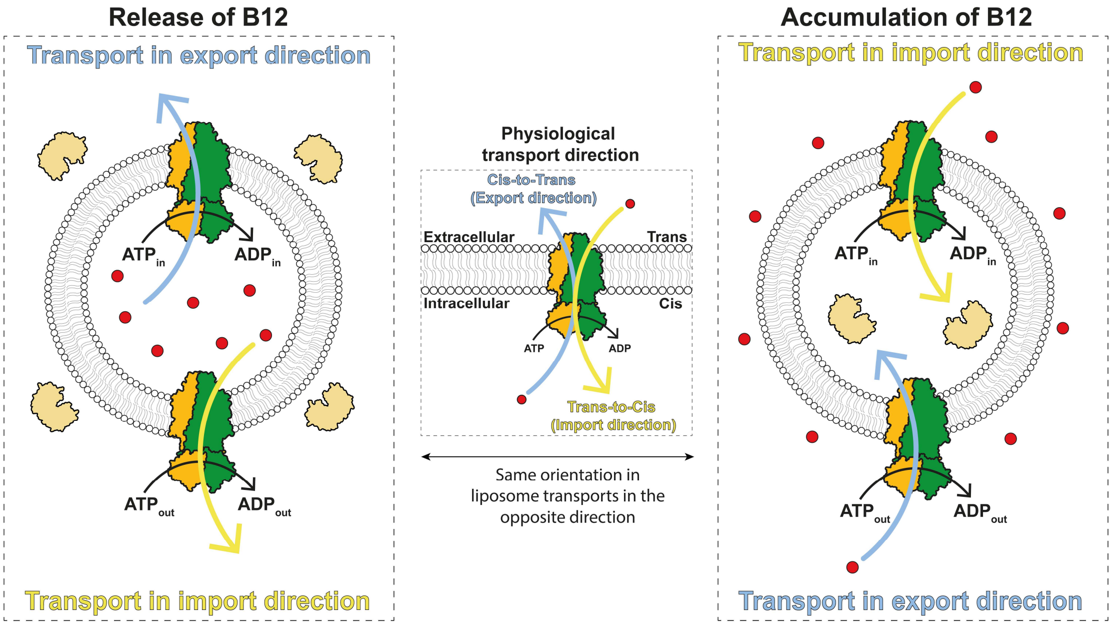

Supplementary Figure 4: Schematic representation of the fluorescence-based transport assays. In the fluorescence-based release assay, cobalamin is encapsulated inside the lumen of the proteoliposomes. Transport by the different oriented proteins can be triggered separately by external Mg-ATP for transport in the import direction, luminal Mg-ATP for transport in the export direction and Mg-ATP on both sides of the membrane for transport by both populations inside the membrane. In the reversed fluorescence-based accumulation assay, the sensor is encapsulated inside the lumen of the proteoliposomes, while cobalamin is added to the external buffer. Transport by the different oriented proteins can be trigged separately by luminal Mg-ATP for import activity, external Mg-ATP for transport in the export direction and Mg-ATP on both sides of the membrane for transport by both populations inside the membrane. The liposome membrane is shown as grey circle, the sensor in yellow, cobalamin as red dots and the two $BacA_{Mm}$ populations in green-orange. Export and import directions are indicated by blue and yellow arrows respectively.

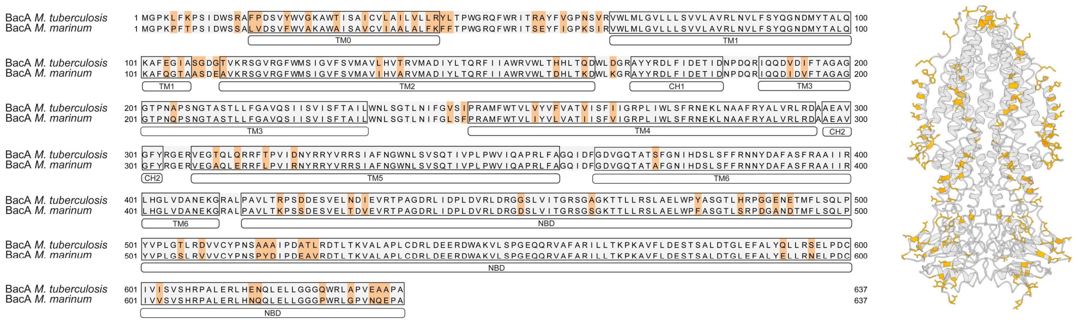

Supplementary Figure 5: Sequence alignment of $BacA_{Mt}$ and $BacA_{Mm}$. The sequences alignment was performed with Clustal Omega and was visualized using Jalview. The structural elements of the protein are indicated in bars below the sequence as: transmembrane regions (TM), coupling helices (CH) and nucleotide binding domains (NBDs). The differences between $BacA_{Mt}$ and $BacA_{Mm}$ are highlighted in orange and are mapped on the structure of $BacA_{Mt}$ (PDB: 6TQF).

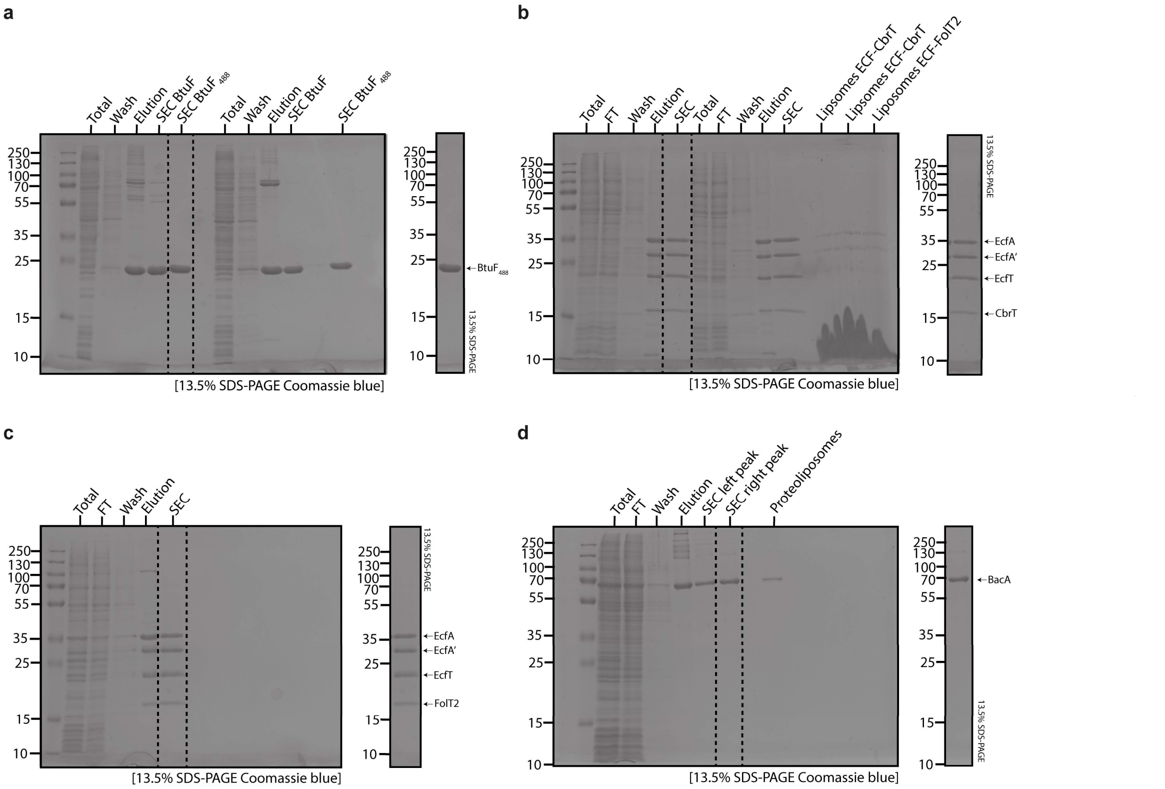

Supplementary Figure 6: uncropped SDS-PAGE gels. Uncropped images of SDS-PAGE gels that were used for Supplementary Fig. 1. **a** $BtuF_{488}$, **b** ECF-CbrT, **c** ECF-FolT2, **d** $BacA_{Mm}$.

Table 1: Bacterial strains used in this study.

Expression host	Construct	Organism of origin
E. coli MC1061	pBAD18+6_BtuF$_{Ec}$_ΔCys_D141C_His8	*E. coli*
E. coli MC1061	pBXC3H_ BacA$_{Mm}$	*M. marinum*
E. coli MC1061	p2BAD_Ecf_CbrT_Ld_ΔCys_His8	*L. delbrueckii*
E. coli MC1061	p2BAD_Ecf_FolT2_Ld_His8	*L. delbrueckii*
E. coli ΔFEC	pBXC3H_ BacA$_{Mm}$	*M. marinum*
E. coli ΔFEC	p2BAD_Ecf_CbrT_Ld_ΔCys_His8	*L. delbrueckii*
E. coli ΔFEC	p2BAD_Ecf_FolT2_Ld_His8	*L. delbrueckii*

Table 2: Primers used in this study.

Primer name	Sequence 5'-3'
Fw_Fx_BacA	ATATATGCTCTTCTAGTGGCCCGAAGCCGTTTAC
Rv_Fx_BacA	TATATAGCTCTTCATGCGGCCGGTGCCGGCTCCTG
Fw_EcfA'_C168A	GATCATCGCTTTAGATGAGCCGGCAGC
Rv_EcfA'_C168A	CATCTAAAGCGATGATCTCCGGCTC
Fw_EcfT_C252S	CTACTCCCTGCTGTTGACAATTTTG
Rv_EcfT_C252S	GCAGGGAGTAGGCTACTGGAATC
Fw_CbrT_C22S	GCTGACTGCGATGTCCGTGGTTTTGCGG
Rv_CbrT_C22S	CCGCAAAACCACGGACATCGCAGTCAGC
Fw_CbrT_C86S	GCCTACGCGGCCTCTGCTTTGACCG
Rv_CbrT_C86S	CGGTCAAAGCAGAGGCCGCGTAGGC
